# Translating Molecular Profiling of Soft Tissue Sarcomas into Daily Clinical Practice

**DOI:** 10.3390/diagnostics11030512

**Published:** 2021-03-14

**Authors:** Celine Jacobs, Lore Lapeire

**Affiliations:** 1 Department of Medical Oncology, Ghent University Hospital, 9000 Ghent, Belgium; celine.jacobs@uzgent.be; 2 Cancer Research Institute Ghent (CRIG), Ghent University, 9000 Ghent, Belgium

**Keywords:** molecular profiling, sarcoma, KIT, PDGFRA, NR4A3, MDM2, CDK4, ALK, PD-1, NTRK

## Abstract

Soft tissue sarcomas are a group of rare mesenchymal tumors with more than 70 subtypes described. Treatment of these subtypes in an advanced setting is mainly according to a one-size-fits-all strategy indicating a high unmet need of new and more targeted therapeutic options in order to optimize survival. The introduction of advanced molecular techniques in cancer has led to better diagnostics and identification of new therapeutic targets, leading to more personalized treatment and improved prognosis for several cancer types. In sarcoma, a likewise evolution is seen, albeit at a slower pace. This manuscript describes how in the past years advanced molecular profiling in soft tissue sarcomas was able to identify specific and often pathognomonic aberrations, deferring standard sarcoma treatment in favor of more targeted treatment from an oncologist’s point of view.

## 1. Introduction

Soft tissue sarcomas (STS) are rare mesenchymal tumors arising everywhere in the body. Although more than 70 subtypes exist, a one-size fits-all strategy is mostly applied when treating STS patients. For localized disease, the treatment is mainly surgical resection often combined with neoadjuvant or adjuvant radiotherapy. The role of chemotherapy in a curative setting is highly debatable and only used in selected cases [1]. In locally advanced and metastatic disease, chemotherapy remains the backbone of STS treatment. For decades, anthracyclins were the first-line treatment of choice with a marginal response rate of about 15% [2]. Adding ifosfamide to anthracyclines can boost the response rate to 20–25% but at the cost of higher toxicity and, therefore, they are only suitable for fit patients [3]. Gemcitabine-based combinations can reach similar responses to anthracyclins, while other second line treatment options in monotherapy like ifosfamide, pazopanib, trabectedin, dacarbazine and eribulin, barely reach response rates of 10%, indicating need for new and potent therapies [4].

The introduction of more advanced molecular techniques like fluorescence in situ hybridization (FISH), array comparative genomic hybridization (aCGH), whole genome sequencing and RNA-sequencing, has led to a tremendous improvement in both subtyping and treating several cancer types [5]. One of the finest examples is the molecular profiling of nonsmall cell lung cancer (NSCLC), where the discovery of gene alterations in *EGFR*, *ALK*, *ROS1* and *BRAF* not only led to the division of NSCLC in different subcategories with their respective prognoses, but also to the identification of specific druggable targets and a marked improvement in treatment options and survival [6].

In sarcomas, a likewise evolution is seen, albeit at a much slower pace. At the moment, the use of molecular techniques in sarcomas mainly leads to a further diversification of the different sarcoma subtypes. For example, due to advanced molecular profiling, Ewing-like sarcomas can now be subdivided into three main categories with distinct morphological and clinical features: round cell sarcomas with a translocation between *EWSR1* and non-ETS family members, CIC-rearranged sarcomas and BCOR-rearranged sarcomas [7]. However, despite these advances in diagnostic characterization, they have not led to therapeutic consequences, as the treatment of Ewing-like sarcomas remains similar to the treatment of a classic Ewing sarcoma.

The importance of molecular pathology in the further unravelling of sarcoma subtypes can never be overestimated, but for the sarcoma patient its true power lies in its capability of identifying new therapeutic targets and optimizing sarcoma treatment. In this manuscript we describe some interesting examples of how in the past years molecular profiling has been able to shift systemic treatment from the standard, often one-size-fits-all, management towards precision oncology in locally advanced or metastatic soft tissue sarcomas (STS). We mainly focus on those molecular discoveries that (1) pointed towards putative benefit of drugs already used in other cancer types and available for sarcoma patients through reimbursement, specific medical need programs or compassionate use, and (2) stimulated pharmaceutical companies to develop a new targeted drug or treatment that has almost, or already, reached market authorization. 

## 2. KIT and PDGFRA Mutations in Gastro-Intestinal Stromal Tumors (GIST)

A GIST is the most common mesenchymal neoplasm of the gastro-intestinal tract. Seventy to eighty five percent of GISTs have gain-of-function mutations in *KIT* and 10–15% gain-of-function mutations in *PDGFRA* [8]. The small remaining part of GISTs have a deficiency in succinyl dehydrogenase (SDH) activity, a *NF1* mutation, a *BRAF* V600E mutation or none of the above [9]. The discovery that GISTs are mainly dependent on KIT or PDGFRA receptor tyrosine kinase signaling was not only of great diagnostic, but also therapeutic value. GISTs are the only sarcoma subtype that can be treated with reimbursed targeted therapy in an advanced setting up to four lines, specifically targeting KIT/PDGFRA tyrosine kinase receptors (first line imatinib, second line sunitinib and third line regorafenib). EMA (European Medicines Agency) approval for the fourth line ripretinib/avapritinib is expected in 2021. For a recent and comprehensive review about this topic we refer to Li et al. [10]. The use of advanced molecular techniques in GIST did not only identify putative therapeutic targets but also primary or secondary resistance mechanisms which can have therapeutic consequences. Wild-type GIST and GIST with the *KIT* exon 9 mutation or *PDGFRA* exon 18 D842V mutation, are associated with primary resistance against imatinib. In the case of a *KIT* exon 9 mutation, twice the standard dose of imatinib is required to achieve acceptable response rates [11]. Secondary resistance to imatinib is mainly due to the development of new mutations in *KIT* exon 13, 14, 17 or 18 upon imatinib treatment [10]. Switching to a different tyrosine kinase inhibitor targeting KIT/PDGFRA, as indicated above, is the best strategy to overcome secondary resistance. 

## 3. NR4A3 Translocation in Extraskeletal Myxoid Chondrosarcoma (EMCS)

EMCS is a subtype of soft tissue sarcoma usually arising in the proximal extremities and limb girdles. It occurs almost exclusively in adults with a median age of 50 years [12]. In more than 90% of cases, a NR4A3-fusion can be found making it a diagnostic hallmark for EMCS. The most common translocation partners are *EWSR1* and, in a much lower frequency, *TAF15*, while *TCF12*, *TFG* and *HSPA8* are reported anecdotally [12,13]. For inoperable, advanced and metastatic disease, a systemic treatment is necessary in the case of progressive disease. EMCS is known to have a rather modest sensitivity to conventional chemotherapy based on anthracyclins, ifosfamide, dacarbazine or trabectedin [14]. Interestingly, antiangiogenic therapy is reported as a valuable alternative treatment option, especially in EMCS harboring the typical EWSR1-NR4A3 translocation. The antitumor activity of sunitinib in EMCS was first described in 2012 by Stacchiotti et al. in two patients with progressive metastatic EMCS [15]. This was later confirmed in a case series of 10 EMCS patients treated continuously with 37.5mg/day sunitinib for progressive metastatic disease. Six Response Evaluation Criteria in Solid Tumors (RECIST) partial responses, two stable diseases and two progressive diseases were noted. Intriguingly, all patients with an objective response (OR) had *EWSR1* as translocation partner for *NR4A3,* while the two cases with progressive disease harbored a TAF15-NR4A3 translocation [16]. A phase 2 trial with the antiangiogenic drug pazopanib for treatment of advanced EMCS showed similar results. Four out of 22 evaluable patients achieved a RECIST partial response, and all four were positive for the typical EWSR1-NR4A3 translocation. Of the three patients with TAF15 as translocation partner, none showed a response [17]. Of note, the median progression-free survival of the EWSR1-NR4A3-positive group was markedly better than that of the TAF15-NR4A3 cases, indicating that TAF15-NR4A3 translocated EMCS is a more aggressive subgroup with insensitivity to antiangiogenic treatment.

## 4. MDM2 and CDK4 Amplification in Liposarcoma (LPS)

LPSs represent about 20% of all soft tissue sarcomas, thereby being the most common subtype [18]. LPSs are divided in different subcategories with an intermediate to malignant behavior. Well-differentiated and dedifferentiated liposarcoma (WD- and DD-LPS) are two subtypes of LPS that are genetically related through an amplification of the oncogene murine double minute 2 (*MDM2*). In fact, *MDM2* amplification is pathognomonic for diagnosing WD- and DD-LPS, making it an interesting potential therapeutic target [19]. A proof-of-mechanism study reported on activation of the P53 pathway and subsequent decreased tumor cell proliferation when patients with a resectable MDM2-amplified WD- and DD-LPS were treated with RG7112, a small molecule MDM2-antagonist, in a neoadjuvant setting (window of opportunity) [20]. Later on, a phase I study of SAR405838, a novel human double minute 2 (HDM2) antagonist, in patients with advanced solid tumors, showed only a limited activity with no observed OR [21]. In the maximum tolerated dose (MTD) cohort of this study, only patients with a DD-LPS were allowed. Genetic analysis of the available tumor baseline biopsies indicated that all tumors harbored wild-type *TP53*. Interestingly, genetic analysis of cell-free DNA in plasma samples of these patients showed the appearance of *TP53* mutations upon treatment with the HDM2 antagonist, indicating a possible mechanism of required resistance to HDM2 inhibition, and most probably explaining the observed limited activity [22]. At present, the therapeutic value of MDM2 or HDM2 inhibition in WD- or DD-LPS is unclear. Currently, only two clinical trials with a MDM2 inhibitor are open for recruitment. The first one is a phase 1b trial to evaluate the safety of combining the MDM2 inhibitor AMG-232 with radiotherapy in a neoadjuvant setting for soft tissue sarcomas (NCT03217266). The second is a phase 1b/2 study of the MDM2 inhibitor APG-115 in combination with pembrolizumab in patients with TP53 wild-type and MDM2-amplified liposarcomas, amongst other cancer types (NCT03611868).

Next to the canonical *MDM2* amplification in WD- and DD-LPS, almost 90% of cases also harbor a cyclin-dependent kinase 4 (*CDK4*) amplification, identifying CDK4 as an interesting potential therapeutic target, especially given the excellent results of CDK4/6 inhibitors in hormone receptor-positive, human epidermal growth factor receptor 2-negative breast cancer [23,24]. The authors conclude that CDK4 targeting in WD- and DD-LPS elicited a significant activity with durable disease stabilization. Although the CDK4/6 inhibitor Palbociclib is a category 2A consideration in retroperitoneal WD- and DD-LPS in the national comprehensive cancer network (NCCN) guidelines, there is currently no reimbursement of a CDK4/6 inhibitor in this, or other, LPS indication. However, several clinical trials are open for recruitment investigating a CDK4/6 inhibitor in monotherapy, or in combination therapy, in LPS and other sarcoma subtypes.

## 5. ALK Expression in Inflammatory Myofibroblastic Tumors (IMTs)

IMTs are rare soft tissue tumors, most frequently diagnosed in children and adolescents, but they can occur in adults as well. According to the WHO classification they are graded as intermediate tumors with local recurrence, being the most frequent form of relapse, and formation of metastases being rather rare. Surgery is the backbone of treatment in these patients if it is feasible, as IMTs are not chemo- or radiosensitive. About 50% of IMTs harbor an anaplastic lymphoma kinase (*ALK*) mutation on chromosome 2p23, which leads to excessive *ALK* expression [25]. The *ALK* mutation can be screened by immunohistochemistry (IHC) but has to be confirmed by FISH. *ALK* expression does not seem to have a prognostic impact in IMT, although data are conflicting [25,26]. In *ALK*-mutated non small cell lung cancer (NSCLC), the introduction of targeted therapy with tyrosin kinase inhibitors (TKI) directed against *ALK* was a breakthrough in the treatment of these patients. Crizotinib, which inhibits *ALK*, *MET*, *RON* and *ROS-1*, was one of the first ALK inhibitors which proved effective in *ALK* mutated NSCLC with a positive effect on progression free survival (PFS) [27,28]. In accordance, crizotinib has been used in patients (adults and children) with *ALK*-positive IMTs [26,28,29]. Although IMT is a rare tumor, there have been two small trials which examined the effect of crizotinib: one in a pediatric population in 2013 by Mossé et al., and one in an adult population in 2018 by Schöffski et al. [29,30]. 

In the study by Mossé et al., three of seven patients with *ALK*-positive IMT had an OR [30]. In the study by Schöffski et al., objective responses were seen in six of 12 patients with *ALK*-positive IMT and in only one of seven patients with *ALK*-negative IMT [29]. Both studies support all the case reports describing responses in *ALK*-positive IMT patients treated with crizotinib, as described in the review from Theilen et al. In this review, various case reports showed a complete response (CR), others only partial response (PR) or stable disease (SD) [31]. For the future, it should be interesting to keep reporting the effect of crizotinib in these patients and, if there is progression under crizotinib, to seek for resistance mechanisms as described in NSCLC. Today, there are second (for example ceritinib) and third generation (for example lorlatinib) ALK inhibitors available for NSCLC, so if a patient with *ALK*-positive IMT fails to respond to crizotinib, or if there is progression under crizotinib, these drugs could be an option to overcome the underlying resistance mechanisms. An example of this was reported by Mansfield et al., who described a patient with IMT who responded to ceritinib (second generation ALK inhibitor) with a partial response, after progression under crizotinib [32]. Wong et al. described a partial response to lorlatinib (third generation) in a patient with *ALK*-positive IMT, who previously received prednisolone, eretrectinib and adriamycin-ifosfamide. We note that this patient did not receive a first or second generation ALK inhibitor [33]. 

## 6. BRAF Mutation in Soft Tissue Sarcoma (STS)

The mitogen-activated protein kinase (MAPK) pathway is a well-known pathway that leads to development of several targeted agents like vemurafenib, dabrafenib, trametinib, etc. The best known downstream signaling pathway is the RAS/RAF/MEK/ERK pathway. Mutations in *RAS* and *RAF* are the main cause of oncogenic activation of the MAPK pathway. In daily practice, the *BRAF* V600E mutation is best known, for example in patients with metastatic melanoma who respond very well to BRAF/MEK inhibitors. However, *BRAF* mutations are not limited to melanoma patients. They have also been described in other cancer types, like undifferentiated thyroid cancer, colon cancer and also in STS. The presence of the *BRAF* mutation in STS is rare, explaining why treatment with BRAF/MEK inhibitors in STS has not been investigated in a clinical trial. However, there are several case reports of the presence of the *BRAF* mutation in different sarcoma subtypes, like malignant peripheral nerve sheath tumors (MPNST), GIST and Ewing sarcoma. These cases, however, differ substantially in the given treatment (type of drug, combination of BRAF/MEK inhibition versus monotherapy) [34]. Protsenko et al. reported a case of a patient with a clear cell sarcoma who responded very well to vemurafenib [35]. Watanabe et al. described two patients with metastatic synovial sarcoma who had a BRAF V600E mutation. One patient received dabrafenib and trametinib, with a partial response for 7.5 months [36]. 

We know from data of melanoma patients that combining BRAF and MEK inhibitors is better than BRAF-directed monotherapy, due to resistance of the tumor cells [34,37,38,39]. Ideally, trials with BRAF and /or MEK inhibitors, or other combinations, will be performed in the future, but due to the rarity of STS and the rarity of the *BRAF* mutation in STS, this will be extremely difficult. Setting up basket trials with NGS (next genome sequencing) testing, in which STS patients can be included in a therapeutic arm according to their NGS results, could provide a solution. 

## 7. TRK Fusion-Positive STS

Tropomyosin receptor kinases (TRKs) are transmembrane receptor tyrosine kinases, encoded by neurotrophic tyrosine receptor kinase 1, 2 and 3 genes (*NTRK 1*, *NTRK2* and *NTRK3*). Investigation showed that oncogenic *NTRK* gene fusions occur due to genomic rearrangements which can result in tumorigenesis. *NTRK* fusion mutations are highly represented in some tumor types like infantile fibrosarcoma (IFS) and secretory breast cancer, but in most tumors their frequency is rather low, as is the case for soft tissue sarcomas. Pan-TRK inhibitors have been developed during the recent years with very good observed responses [40].

The first difficulty is how to screen for *NTRK* fusions, since there are multiple techniques available such as IHC, FISH and RNA- and DNA-based NGS assays. The European Society of Medical Oncology (ESMO) group has published guidelines about *NTRK* fusion screening in specific situations. If the histologic tumor type is known to harbor a high frequency of NTRK gene fusions, the best option is to use FISH, Reverse Transcription- Polymerase Chain Reaction (RT-PCR) or RNA-based NGS. If the histologic tumor type is not known to harbor a high frequency of *NTRK* gene fusions, the panel suggests screening with IHC and, if positive, to seek confirmation with NGS techniques or, if NGS techniques are easily available, to use NGS preferably including RNA testing [41]. 

Larotrectinib is a highly selective pan-TRK inhibitor initially tested in a phase I trial in patients regardless of their *NTRK* fusion gene status [42]. In 2018, Drilon et al. published the first pooled analysis of safety and efficacy results of larotrectinib in *NTRK* fusion-positive cancer patients who were treated in a phase 1 study (adults, eight patients), a phase 1–2 study (children, 12 patients) and a phase 2 study (adolescents and adults, 35 patients) [43]. Taken together, a total 55 cancer patients were included, of which 20 were diagnosed with an STS (seven IFS, three GIST, three spindle-cell tumors, two MPNST, two myopericytoma, two undifferentiated pleomorphic sarcoma (UPS) and one infantile myofibromatosis), with an ORR of 75%. Responses were seen regardless of tumor type, age or fusion status. Thirteen percent had a CR and 62% had a PR. The median duration of response and median PFS (mPFS) had not been reached after, respectively, 8.3 months and 9.9 months follow-up. The adverse events (AE) were tolerable, with mainly grade 1–2 events [43]. Based on these results larotrectinib was approved by the US Food and Drug Administration (FDA) and theEMA) for advanced solid tumors with *NTRK* gene fusion. Hong et al. published in 2020 an expanded pooled efficacy analysis of 159 patients with TRK-fusion positive cancer who were treated with larotrectinib. The updated results showed an OR of 79% with a CR in 16% and a median duration of response of 35.2 months [44]. It is also important that patients with intracranial disease showed responses as well, which confirms previous reports [44,45]. Entrectinib is another pan-TRK inhibitor that proved its effect in phase I/II studies, of which pooled results of 54 adults with *NTRK* fusion-positive solid tumors were published by Doebele et al. in 2020. At a median follow-up of 12.9 months, 57% had an objective response (7% CR and 50% PR). Entrectinib has also been approved by the FDA for *NTRK* fusion-positive solid tumors [40,46]. 

As we see with all targeted therapies, tumors can have primary resistance or can develop acquired resistance to these drugs [40,44]. Secondary resistance, due to several mutations, has been described and next-generation TRK inhibitors are under development [40,46]. 

TRK inhibitors are quite unique in their tumor-broad efficiency and can be of high value in STS, but we do have to emphasize that *NTRK* fusion mutations are rare in most STS. We await published data of sarcoma-specific results to have more insight in the role of TRK inhibitors in this heterogenous group, currently studied in an advanced setting, but possibly in the future also in a neoadjuvant setting.

## 8. Immunotherapy and PD-1/PD-L1 in STS

Several immunotherapeutic agents, like antiprogrammed cell death protein 1 (anti-PD-1) antibodies, antiprogrammed cell death protein ligand 1 (anti-PD-L1) antibodies and anticytotoxic T-lymphocyte-associated protein 4 (anti-CTLA-4) antibodies, have shown very good results in tumors like melanoma, renal cell carcinoma and lung cancer, with durable responses never seen before. In STS, the effect of checkpoint inhibitors has also been investigated, together with the value of PD-1/PD-L1 staining. PD-1/PD-L1 expression is not sufficient as a biomarker to predict response on immunotherapy, as we also know from investigations in other tumor types [47,48]. Other markers that may predict the benefit of immunotherapy are not really promising in STS: very few STS are microsatellite-instable (MSI),Tumor-infiltrating lymphocytes (TILs) are often low and the tumor microenvironment of STS is completely different than, for example, in melanoma [49]. As we know, STS is a very heterogeneous group of tumors, with different histologies and characteristics, so it is logical that in this very heterogenous group, probably some tumor types such as UPS could be more immunosensitive than others. 

Recent studies have evaluated the effect of anti-PD-1 antibodies in monotherapy, a combination of anti-PD-1 antibodies and anti-CTLA-4 antibodies and a combination of anti-PD-1 antibodies with chemotherapy or targeted therapy in STS. The SARC028 phase II trial with pembrolizumab monotherapy showed an OR in 7 out of 40 patients: four patients with UPS, two patients with DD-LPS and one patient with synovial sarcoma. There were only two patients who had PD-L1 expression, which were the two UPS patients who responded [50]. Other studies investigated nivolumab monotherapy, ipilimumab monotherapy, ipilimumab-nivolumab, axitinib-pembrolizumab or doxorubicin-pembrolizumab [49]. The combination of axitinib and pembrolizumab (second line) showed an overall response rate of 25% and mPFS of 4.7 months, especially in alveolar soft-part sarcomas (ASPS), which was better than results of the IMMUNOSARC trial, which investigated the combination nivolumab and sunitinib (beyond first line), with an ORR of 11% and PFS of 5.9 months [49,51,52]. Pollack et al. investigated the combination of doxorubicin with pembrolizumab in first line in patients with STS with an ORR of 19% and an mPFS of 8.1 months [53]. In general, it seems that combination therapy is better than immunotherapy monotherapy in STS. Further research is necessary in combination with identification of prognostic markers to predict who can respond to immunotherapy. 

Petitprez et al. developed a new classification of STS based on the composition of the tumor microenvironment (TME). They identified five phenotypes of Sarcoma Immune Classes (SIC) [54]:

A: ‘immune desert’: lowest expression of gene signatures related to immune cells, low vascularization

B: heterogeneous, immune-low profiles 

C: highly vascularized (high expression of endothelial-cell-related genes)

D: heterogeneous, immune-high profiles

E: immune-high and TLS-high (tertiary lymphoid structures)

These five SICs were significantly associated with prognosis: group D and E had the best overall survival (OS), and group A had the worst OS. The investigators also studied the correlation between these SICs and response to immunotherapy in the SARC028 trial and found that SIC E tumors were associated with the best response to immunotherapy. The mPFS was also better for SIC E tumors compared to SIC A or B tumors [54]. Petitprez et al. showed interesting data, which can definitely help to determine immunosensitivity of STS based on this new classification instead of the classic immunohistological classification [54]. Future trials should try to implement this new classification system in combination with the histological classification and NGS results, which will result in truly personalized treatment. 

## 9. Colony-Stimulating Factor 1 (CSF1) in Tenosynovial Giant Cell Tumor (TGCT)

TGCT is a rare neoplasm involving the joint synovia, bursae and tendon sheath. Two types are described based on their biological behavior: localized TGCT or diffuse TGCT. Localized TGCT is the most frequent form (80–90%), occurring most frequently in the digits and has a low recurrence rate. Diffuse TGCT, otherwise known as pigmented villonodular synovitis (PVNS), is less frequent (10–20%), more aggressive, more destructive, involves large joints (ankle, elbow, knee, hip) and has a higher recurrence rate [55,56]. 

In 63–77% of patients with TGCT, a minority of the neoplastic tumor cells show a *CSF1* translocation, with *COL6A3* being the most common fusion partner. This translocation leads to overexpression of CSF1 attracting CSF1R-bearing inflammatory cells which form the mass of the tumor [56,57,58]. 

The standard of care is complete resection, which is however often difficult to achieve in diffuse TGCT. If not completely resected the recurrence rate is obviously high, leading to further destruction of the joint [55,56,58].

Pexidartinib is a highly selective tyrosine kinase inhibitor (TKI), targeting and inactivating the CSF1 receptor. Pexidartinib also targets KIT and FLT3-ITD [56,58].

In 2019, Tap et al. published the ENLIVEN study, a phase 3 randomized double-blind placebo-controlled study, to assess the efficacy of pexidartinib in patients with histologically confirmed TGCT with inoperable, symptomatic, advanced disease. The first part was a double-blind phase with 120 patients, of which 61 patients received pexidartinib. The second part was an open label part, in which patients of the placebo group could cross over to receive pexidartinib (*n* = 30). At week 25 there was an ORR of 39% in part 1, and 30% in part 2, with a best ORR of 53% in both groups. Pexidartinib also significantly increased the range of motion of the affected joint and the physical functioning [56]. 

Gelderblom et al. published a pooled analysis of the phase 1 study and the ENLIVEN trial in 2020, with a total of 130 patients. Sixty percent achieved an OR (CR or PR) by RECIST, and 26% achieved a CR, with a median time to response of 3.4 months and a median duration of treatment of 19 months. We remark that 92% showed a response by 18 months [59]. 

The main concern regarding the use of pexidartinib is liver toxicity, with two fatalities reported across all patients in the pexidartinib trials. Pexidartinib is currently approved by the FDA, but only available through a risk evaluation management system program. It is not EMA approved, due to the liver toxicity. 

New drugs for TGCT are being tested in clinical trials, for example emactuzumab, a humanized monoclonal antibody which inhibits CSF1R activation. A phase 1 trial in 63 patients showed an ORR of 64% after two years. The most frequent AEs were pruritus, asthenia and oedema [59]. Further results of upcoming trials with this drug are awaited. 

## 10. Loss of SMARCB1 (INI1) in Epithelioid Sarcoma (ES)

ES represents less than 1% of all adult STS and 4–8% of childhood non rhabdomyosarcomatous STS. Clinically, there are two distinct types: distal-type ES characterized by a superficial, slowly growing mass at the distal part of the (mostly upper) extremities of mainly adolescents and young adults, and proximal-type ES developing a large, deeply located and more infiltrative mass at the proximal lower extremities and pelvic area (perineal, genital) in a young to middle-aged population [60]. ES is one of the rare STS subtypes that shows lymph node dissemination. In 90% of cases, the diagnosis of ES is typically made by demonstrating the complete loss of *SMARCB1*/INI1 [61]. INI1 is coded by *SMARCB1* and functions as a tumor suppressor gene. It is a member of the SWI-SNF chromatin-remodeling complex, and loss of INI1 leads to uncontrolled cellular growth and neoplastic transformation [62]. INI1 inactivation leads to overactivation of polycomb repressive complex 2 (PRC2), stimulating the methylation of histones, cell proliferation and silencing of genes responsible for differentiation [63]. Methyltransferase EZH2 is a subunit of PRC2, and inhibition of EZH2 shows regression of malignant rhabdoid tumor xenografts [64]. 

Tazemetostat is an oral selective EZH2 inhibitor studied in advanced ES with loss of *SMARCB1*/INI1 in an international, open-label, phase 2 basket trial. Nine out of 62 patients showed an objective response, while in 16 patients stable disease was noted [65]. 

Based upon these results, tazemetostat received accelerated approval in the USA for the treatment of adults and adolescents aged 16 years or older with locally advanced, or metastatic, ES not eligible for complete resection. Outside of the USA, ES patients can be treated with tazemetostat through an Expanded Access Program. Currently, a phase 1b/3 clinical trial investigating tazemetostat in combination with doxorubicin as frontline therapy for advanced ES is open for recruitement (NCT04204941). 

## 11. NY-ESO-1 in Synovial Sarcoma (SS)

Synovial sarcoma (SS) is a well-known STS subtype mostly located in the deep soft tissue of the extremities but has been described in numerous other regions and organs in the human body. It can occur at any age but mostly before the age of 50 years. SS is typically diagnosed by an SS18 rearrangement [60]. Next to this, the cancer testis antigen NY-ESO-1 is highly expressed in SS and implicated in tumor cell proliferation. It can stimulate both antibody and T cell responses [66].

LV305 is a dendritic cell-targeted lentiviral vaccine that encodes NY-ESO-1. After injection, it is processed by dendritic cells, which present the NY-ESO-1 peptides on their cell surface by MHC molecules. This triggers interaction with T cells and can cause an immune response in the tumor [66]. A phase 1 trial tested LV305 in heavily pretreated patients, including NY-ESO-1 positive sarcoma. Fifty-four percent of the sarcoma patients had a SD. One patient with synovial sarcoma had a PR, which was ongoing at three years follow-up [66,67]. 

CMB305 is a regimen which adds vaccine G305 to the LV305 vaccine, to boost the latter. In a phase 1 trial in patients with metastatic disease, including 25 sarcoma patients, the CMB305 regimen was not significantly better than LV305 monotherapy in terms of PFS, although the measured anti-NY-ESO-1 T cells and antibodies were significantly higher [66,68]. Later, a phase III trial was conducted to evaluate the effect of CMB305 as first line therapy in metastatic or locally advanced synovial sarcoma (NCT03520959). Unfortunately, the trial was closed due to slow accrual. 

In 2019, Chawla et al. published the abstract of a phase II trial which investigated the combination of atezolizumab with or without CMB305 in patients with recurrent, metastatic or locally advanced NY-ESO-1 positive sarcoma (SS and myxoid round cell liposarcoma). There was no significant difference in PFS and OS between the two treatment arms. Still, the authors concluded that further investigation with this combination is warranted as more patients in the combination group had a PR and a higher level of anti-NY-ESO-1 immune response, and numerically better outcomes in PFS and OS were observed [69]. 

A different way of targeting NY-ESO-1 in SS is using adoptive T-cell therapy. This technique encompasses the transfer of T cells that are genetically engineered to recognize NY-ESO-1. The first clinical trial in which advanced synovial sarcoma patients were treated with NY-ESO-1-specific T cell receptor (TCR) T cells showed an objective response in four out of six patients [70]. Another study with NY-ESO-1-specific TCR T-cell therapy in metastatic synovial sarcoma showed an objective response in sic out of 12 patients [71]. In both studies, responses were durable for several months, indicating an interesting treatment option for SS.

## 12. Conclusions and Future Perspectives

In the past years, the introduction of advanced molecular techniques has led to a marked improvement of sarcoma diagnostics and, in some specific sarcoma subtypes, also to a significant therapeutic advantage. The use of these techniques in daily clinical practice sometimes leads to the identification of druggable targets. However, the implicated drugs are often not reimbursed or available for sarcoma patients. To move further along the path of precision oncology in sarcomas in the future, we recommend implementing genomic screening and RNA-sequencing in clinical (basket) trials to ensure proper documentation and reporting of new promising therapeutic targets. The MULTISARC trial (NCT03784014), the pediatric MATCH screening trial (NCT03155620) and the RNASARC trial are three excellent examples of currently recruiting clinical trials that integrate molecular profiling of the tumor in the treatment selection of matching readily available drugs for the participating sarcoma patients. The results of these and other similar clinical trials will show us if molecular profiling in sarcomas can help identify new promising therapeutic targets and expand the therapeutic options in daily clinical practice. 
